# SRC-2 Coactivator: a role in human metabolic evolution and disease

**DOI:** 10.1186/s10020-020-00168-0

**Published:** 2020-05-14

**Authors:** Bert W. O’Malley

**Affiliations:** Department of Molecular and Cellular Biology, Baylor College of Medicine, 1 Baylor Plaza, Houston, TX 77030 USA

**Keywords:** SRC-2, Coactivators, Metabolism

## Abstract

The large family of transcriptional coactivators originated with the cloning of the subfamily of Steroid Receptor Coactivators (SRC-1,2,3). These 3 coactivators serve as primary ‘master genes’ to direct the coordinate transcription of multiple genes required for physiological goals in cells, specifically, carbohydrate, lipid, or anabolic growth metabolisms. SRC-2 is of special interest in terms of lipid metabolism and energy accrual and is the topic of a collection of our research discoveries and publications described in this Perspective.

## Background

The aims are to summarize our lab’s efforts in unraveling the function of a major coactivator, SRC-2, in metabolism and energy accumulation.

## Main text

In reflecting on our work over the past decade on transcriptional coactivators, I have been impressed with data that marks the Steroid Receptor Coactivator-2 (SRC-2) molecule as an important regulator of life, reproduction, and the geographic spread of early humans throughout the world. SRC-2 is a member of the SRC-family of 3 coactivator genes (SRC-1, − 2,and − 3). My attention drifted to coactivators because of our primacy in these molecules and my prior recognition of these enhancer-dependent proteins as ‘masters of the genome’ whereby they coordinate the expression of large arrays of genes to produce a physiologic goal (O’Malley, 2010). The SRC-coactivators are at the nexus of over 75% of all gene expression. Their genetic regulatory roles, however, are not random. SRC-1 is more dedicated to genes of carbohydrate metabolism, SRC-2 to genes of lipid metabolism, and SRC-3 to anabolic and growth response genes. SRC-2 especially is intriguing to me due to its relevance to food acquisition and energy storage, and to its potential for human population migration.

Considering the amazing migratory spread of humans, as they moved out of Africa and populated the distant regions of the world, food availability was likely the primary obstacle to long distance travel and migration. Not able to carry enough food with them over a long distance, and not having yet developed good ‘farming’ skills, food was an obvious worry for survival. In short, these travelers needed to carry as much high caloric nourishment with them as possible, and the superior choice in terms of its caloric content was body fat. Consequently, their efficiency in intestinal fat absorption and storage became critical to their energy level and survival. Obviously, there were driving metabolic forces for their survival during this period?

I would like to propose that the SRC-2 coactivator, also known as GRIP1 and TIF2 by the labs that cloned the molecule, (Hong et al., 1996; Voegel et al., 1996) is likely one of the foremost forces in our evolutionary process. What might be my reasoning for this consideration? I theorize that in the early evolution of humans, the metabolic efficiency emanating from function of this coactivator molecule was critical for survival because it enhances instestinal fat absorption and bodily storage, providing a repository for later delivery of energy. In early times (~ 100,000 years ago), fat clearly would have been our best natural source of calories (9 cal./g.) for sustenance during migration. Nowadays, however, SRC-2’s molecular efficiency in accumulation and storage of caloric fat is harming inhabitants of the Western world by its resultant obesity, enhanced by the plethora of cheap high calorie food in our diets which predisposes us to obesity and diabetes.

In the past 10 years, we established an interesting ‘big picture’ for the presence and evolutionary value of SRC-2. From separate studies, we found that the SRC-2 coactivator is a bone fide ‘metabolic master gene’ for physiology and lipid metabolism (O’Malley, 2010; Dasgupta et al., 2014). It appears that the actions of SRC-2 encourage obesity and diabetes, as well as promote metabolic changes that favor intracellular fat accumulation and cancer cell survival following metastasis (Chopra et al., 2011a; Dasgupta et al., 2015). It operates in harmony with another ‘master mitochondrial regulator’, PGC-1, whereby they all direct specific and complimentary aspects of lipid and carbohydrate metabolism. PGC-1 has been the subject of multiple reviews and will not be discussed further herein.

We discovered SRC-1, the first member of the SRC- family in 1995 (Oñate et al., 1995). The SRC-family contains three related coactivators; all 3 SRCs are oncogenes when overexpressed; although all are transcriptional regulators of steroid receptors, they also coactivate many other transcription factors implicated in various cell functions. Each of these coactivators act as ‘master regulators’ for the expressions of large banks of genes that must be coordinately regulated to effect major physiological goals. SRC-2 has a specific target gene signature, one which directs efficient metabolism (Fleet et al., 2015; Chopra et al., 2008; Stashi et al., 2014b); these reactions and processes can be grouped into the broad goal of energy accretion, storage and utilization; it also controls our diurnal metabolic rhythms. Importantly, SRC-2 is indispensable for the propagation of animal species due to its critical roles in mammalian reproduction where it functions in embryo implantation and pregnancy (Szwarc & Kommagani, 2014).

SRC-2 regulates a host of reactions in lipid intermediary metabolism. The first hint of a whole body role for the SRC-2 coactivator was the observation of greatly diminished body fat in animals when its coding gene was deleted. We then demonstrated that SRC-2 is necessary for absorption of high energy yielding fats from the diet. It does this by controlling bile release from the liver via regulation of the bile secretory export protein (BSEP) gene; secreted bile flows via the gall bladder into the intestine for fat emulsification and absorption in the ileum (Chopra et al., 2011a). Following its role in fat absorption, it also enhances storage of excess lipid (calories) in an animal’s adipose reserves. These two closely related processes, fat absorption and storage, represent the logical coordination required of a master energy coactivator. Our recent observation that SRC-2 plays an important role in mouse diurnal metabolic rhythm (Chopra et al., 2011b), a process integrally related to eating, activity, and obesity/diabetes, further substantiates it physiologic role.

SRC-2 also has another interesting role in its metabolic oversight of the body. When food is withdrawn during fasting, SRC-2 regulates hepatic glucose output by virtue of its activation of the glucose-6-phosphatase gene (Chopra et al., 2008). This provides immediate short-term glucose to the circulation and brain. Longer term for survival, however, requires the greater caloric availability of cellular fatty acids and cholesterol. The body can first raid its stores of fatty acids, but ultimately coordinated regulation of the synthesis of fatty acids and cholesterol from pyruvate must occur for future supplementation. When eating, glucose levels in blood rise and intracellular glucose can be utilized for re-synthesis of fatty acids and cholesterol. Our recent evidence which reveals that SRC-2 plays a regulatory role in this process by stimulating expression of genes that convert acetyl CoA to cholesterol (HMGCR gene) and fatty acids (SCD1 gene) (Dasgupta et al., 2015), SRC-2 becomes a one-stop shopping item for coordinate regulation of energy accretion and utilization at both the cellular level and whole body level (Stashi et al., 2014a).

It is of interest that this same coactivator, SRC-2, is a key player in pregnancy. In SRC-2 deficiency, infertility ensues due to poor uterine progesterone-dependent decidualization and a failure of blastocyst implantation (Szwarc & Kommagani, 2014; Mukherjee et al., 2006; Fleet et al., 2016). Why is pregnancy linked to a coactivator that directs lipid accretion and adipogenesis? Perhaps it is due to the fact that pregnancy is meant to occur when the female body is well nourished and has a store of fat from a plentiful caloric environment. In this way, the female can survive the rigors of nourishing two humans at the same time, as well as provide resources for offspring.

Although it is not hard to imagine how important this coactivator was for early human nutrition, it also may shorten our modern life span from a completely different point of view. Recent evidence finds SRC-2 to be a commonly overexpressed oncogene in certain types of aggressive cancers. Metastatic lesions of the prostate have a high level of SRC-2 gene amplification and overexpression of the SRC-2 gene (Chopra et al., 2011a); those cells also are loaded with lipids, indicating that in this cancer, it plays an important role in the ‘metastatic and survival’ process. Why would cancers want to overexpress a ‘master lipid metabolic gene’? Certainly, the cancer cell needs a good supply of fatty acids and cholesterol for new membranes required for relentless proliferation and fats from body stores are not easily obtained through the poor circulation and hypoxic inner environment of a cancer mass. Perhaps more importantly, it needs a high level of production and storage of fatty acids for its long distance travel in the body and for survival at metabolic sites until it can live off the new local environment and growth factors – a scenario not unlike the processes required for human migrations described above. I personally find the latter to be most attractive.

## Conclusions

In summary, a potent metabolic regulator such as the SRC-2 coactivator perhaps was once a ‘saving grace’ for early population evolution and survival and colonization of distant lands, but with the change in diet and easy availability of food in much of the developed world, and increasing world obesity in the Western world, it has turned into a ‘sometimes enemy’. What can we do about it? Well, caloric restriction is one way to combat the deleterious consequences of our efficient SRC-2 coactivator. As is well known, caloric restriction in animals is not only an inhibitor of oncogenesis, but it represents the only generally accepted ticket for increased longevity in animals. Caloric restriction is not a prescription for the masses, unfortunately, as it requires a sense of will-power that exceeds the capacity of most humans. Another possible approach to antagonize the metabolic consequences of SRC-2 is to devise mechanisms to inhibit or modulate the actions of this coactivator. Once thought to be nearly impossible, this approach seems now to be plausible. Recent screening and validation assays have led to the development of small molecule inhibitors (SMIs) against the SRC-family of coactivators (Dasgupta & O’Malley, 2014; Song et al., 2016; Lonard & O’Malley, 2012; Wang & Lonard, 2016). Consequently, there is no longer an explicit barrier towards future creation of a small molecule regulatory drug for SRC-2. Such interventions into metabolic pathways will always require caution and consideration of potential negative side-effects. However, coactivators represent a new ‘first in class’ group of targets for pharmaceuticals, and the challenge exists for basic science researchers to fully define the therapeutic feasibility of such newly discovered SMIs that direct their actions against powerful metabolic co-regulators such as SRC-2.

## Data Availability

N/A
